# Ecological strategies and metabolic trade-offs of complex environmental biofilms

**DOI:** 10.1038/s41522-017-0029-y

**Published:** 2017-09-25

**Authors:** Robert Niederdorfer, Katharina Besemer, Tom J. Battin, Hannes Peter

**Affiliations:** 1 Stream Biofilm and Ecosystem Research Laboratory, School of Architecture, Civil and Environmental Engineering, École Polytechnique Fédérale de Lausanne, Lausanne, Switzerland; 2 Department of Limnology and Oceanography, University of Vienna, Vienna, Austria; 3 WasserCluster Lunz, Lunz am See, Austria

## Abstract

Microorganisms aggregated into matrix-enclosed biofilms dominate microbial life in most natural, engineered, and medical systems. Despite this, the ecological adaptations and metabolic trade-offs of the formation of complex biofilms are currently poorly understood. Here, exploring the dynamics of bacterial ribosomal RNA operon (rrn) copy numbers, we unravel the genomic underpinning of the formation and success of stream biofilms that contain hundreds of bacterial taxa. Experimenting with stream biofilms, we found that nascent biofilms in eutrophic systems had reduced lag phases and higher growth rates, and more taxa with higher rrn copy number than biofilms from oligotrophic systems. Based on these growth-related traits, our findings suggest that biofilm succession was dominated by slow-but-efficient bacteria likely with leaky functions, such as the production of extracellular polymeric substances at the cost of rapid growth. Expanding our experimental findings to biofilms from 140 streams, we found that rrn copy number distribution reflects functional trait allocation and ecological strategies of biofilms to be able to thrive in fluctuating environments. These findings suggest that alternative trade-offs dominating over rate-yield trade-offs contribute to the evolutionary success of stream biofilms.

## Complex biofilms: Strategies for success

Analyzing natural biofilms containing many types of bacteria yields insights into microbial strategies for success in complex biofilms. The ecological adaptations and metabolic trade-offs involved in the formation of multi-bacterial biofilms in the environment are not well understood. Researchers in Switzerland and Austria, led by Tom Battin and Hannes Peter at the École Polytechnique Fédérale de Lausanne, performed genetic analysis of biofilms sampled from 140 streams. The biofilms contained hundreds of types of bacteria, unlike the mono-bacterial biofilms examined in many laboratory studies. Genetic analysis techniques revealed a diversity of metabolic strategies that allow bacteria to survive within the rich ecology of natural biofilms. Slow-growing but metabolically efficient bacteria that release more extracellular biofilm components thrive better than those adapted for quick growth alone. The findings significantly improve understanding of biofilm ecology in the natural environment.

## Introduction

Over the last 3.5 billion years, microbial biofilms have undergone multiple evolutionary cycles over which they have developed ecological strategies to exploit diverse niches on Earth.^1,2^ In stream ecosystems, biofilms dominate microbial life, regulate critical ecosystem processes and biogeochemical fluxes that are even of global relevance.^3,4^ The assembly of thousands of bacterial taxa into these complex biofilms and their biodiversity dynamics have been uncovered over the last years.^5–8^ However, the genomic determinants that possibly underlie the formation of complex biofilms and their evolutionary success are not understood.

The availability of cultivation-independent estimates of rrn copy numbers has recently reinvigorated interests in the genomic underpinning of metabolic trade-offs of diverse bacterial communities.^9–11^ This is a major step forward to understand how community-aggregated functional traits give rise to ecological strategies driving adaptations to natural environments.^12–14^ Genes encoding the 5S, 16S, and 23S ribosomal RNA are organized into an operon (rrn) on bacterial genomes. The number of rrn copies ranges between 1 and 15 on bacterial genomes and determines the number of ribosomes that a cell can produce simultaneously.^15–17^ Several studies showed that the number of rrn copies correlates with cellular processes including growth rate, lag time, sporulation efficiency and motility.^9,15,17,18^ There is evidence that bacteria with few rrn copies grow slow but efficiently in terms of resource use, whereas those with higher rrn copy numbers grow rapidly but inefficiently.^10,11,15,19^ The distribution of rrn copy numbers is also indicative of genome streamlining.^18–20^ This not only refers to genome size reduction of bacteria successfully adapted to oligotrophic habitats, but also to small cell size and a preference for a few, broad-specificity transporters over specific energy-intensive transporters.^18–20^ Such trade-offs select for habitat preferences under contrasting regimes of resource availability and ultimately ecological lifestyles can be related to their genomic underpinnings and may explain temporal and spatial population dynamics.^21^

We analyzed the succession of rrn copy numbers in biofilms grown in stream microcosms and containing hundreds of bacterial taxa.^22^ We hypothesized that the distribution of rrn copy numbers in natural biofilms reflects trade-offs between resource use efficiency and growth rate during biofilm formation. We expanded our analyzes to biofilms sampled from 140 streams differing in environmental conditions and including even glacier-fed streams. These latter figure among the most dynamic and extreme stream ecosystems on Earth and yet biofilms thrive in there.^23–25^ We postulate that high environmental fluctuations in these streams favor ecological lifestyles with rapid growth despite resource scarcity.

## Results and discussion

### rrn copy number distribution during biofilm succession

We explored rrn copy numbers of published bacterial sequences (*n* = 2.060.595) from laboratory experiments where we grew biofilms under various trophic and hydraulic conditions^22^ and from various streams^6,24^ (see Methods section). After assignment of rrn copy numbers (see Methods section), we retained operational taxonomic units (OTUs, *n* = 9.676) belonging to 182 genera common to all samples (Supplementary Figure 1). We considered these genera as typical biofilm formers in streams. Based on the differentiation into slow-but-efficient and fast-but-inefficient growth depending on rrn copy numbers,^11,19^ we contrast taxa with low rrn copy number (LCN) by summing up the relative abundances of OTUs with rrn copies ranging from 1 to 3 and taxa with high rrn copy number (HCN) by summing up the relative abundances of OTUs with rrn copies ranging from 4 to 15.

**Fig. 1:**
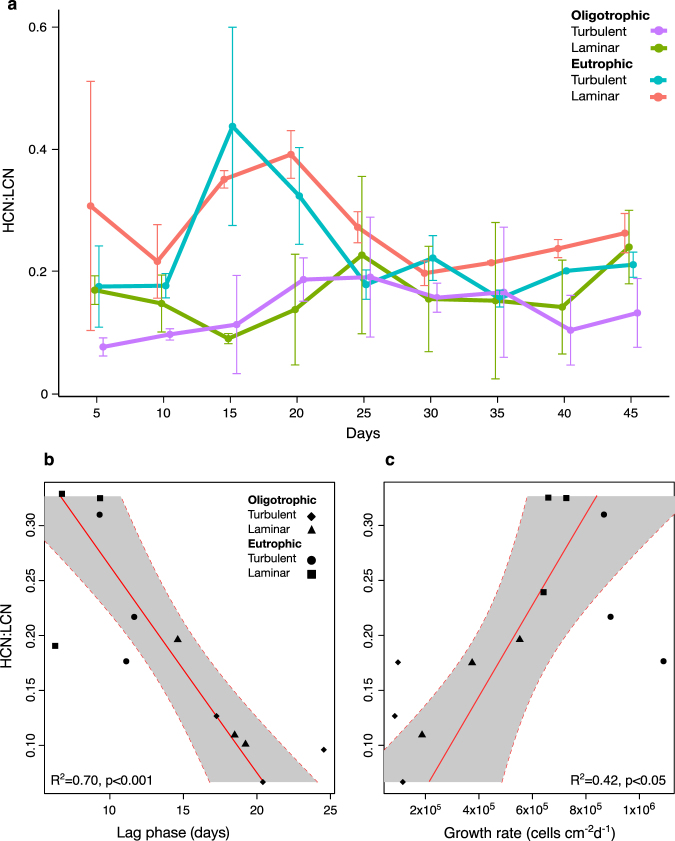
rrn copy number distribution during biofilm succession. Temporal dynamics of average rrn copy number ratio (HCN:LCN) are shown over the course of the two experiments (oligotrophic and eutrophic systems under laminar and turbulent hydraulic conditions) **a**. Error bars display the standard deviation (*n* = 3). The shaded area highlights the initial succession phase. The rrn copy number distribution (HCN:LCN) is related to bulk biofilm growth parameters such as lag phase (*L*) **b** and maximum growth rate (*µ*_*max*_) **c**. The red lines and gray shaded areas represent OLS linear regressions and 95% confidence intervals, respectively

Community average (±standard deviation) rrn copy numbers based on taxa presence was 3.16 ± 0.15 in biofilms grown under oligotrophic conditions and 3.05 ± 0.11 in biofilms grown under eutrophic conditions. Including the relative abundance of taxa, LCN in biofilms from the oligotrophic system were significantly more abundant than in biofilms from eutrophic systems (ANOVA, Tukey’s HSD, *p* < 0.001) as reflected by low HCN to LCN ratios (Fig. 1a). During the initial phase of biofilm formation (up to 25 days), HCN displayed a significantly higher relative abundance in biofilms from eutrophic than from oligotrophic systems (ANOVA, Tukey HSD, *p* < 0.05, Fig. 1a). After this initial phase, ratios of HCN to LCN from both trophic categories converged.

On average, 34.3 ± 0.2 genera contributed to the HCN category while 97.4 ± 4.3 genera contributed to the LCN category in biofilms from the oligotrophic system. In the biofilms from the eutrophic system, HCN and LCN contained 52.5 ± 3.6 and 163.4 ± 16.9 genera, respectively (Supplementary Figure 2). These similar distributions of the number of bacterial genera in HCN and LCN categories in oligotrophic (HCN:LCN genera: 0.35) and eutrophic (HCN:LCN genera: 0.33) environments suggest that differences in diversity did not account for the observed differences in relative abundance of HCN and LCN during biofilm succession.

Next, we related lag phase (*L*) and maximum community growth rate (*µ*_*max*_) as important fitness components of bacterial growth^15^ to rrn copy numbers. Biofilms grown in oligotrophic systems had extended lag phases and lower growth rates compared to those in eutrophic systems.^22^ These growth patterns were reflected by the relative distribution of rrn copy numbers as supported by significant relationships between the average ratio of HCN to LCN and *L* and *µ*_*max*_, respectively (Figs. 1b, c). These findings expand previous studies^19^ on the relationship between rrn copy numbers and growth dynamics of distinct oligotrophic and copiotrophic bacterial species. The relationships between community-level growth dynamics and rrn copy numbers that we found were generated by lumping OTUs at genus level for which rrn copy numbers are available through the rrnDB database.^26^ This underscores the relevance of higher bacterial taxonomic ranks for ecological questions.^27^

### Growth strategies and metabolic trade-offs

**Fig. 2:**
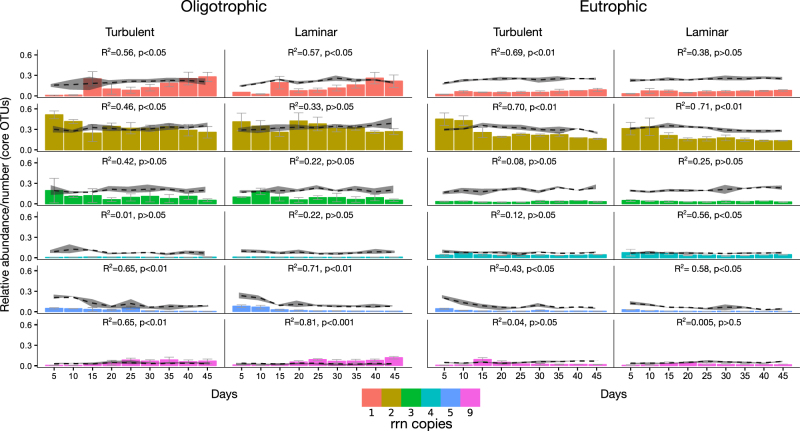
Temporal dynamics of OTUs categorized according to rrn copy number. The bars denote the average relative abundance (*n* = 3) of OTUs with rrn 1–5 and 9. OTUs with rrn 6–8 and >9 were rare and did not contribute to stream biofilm communities originating from oligotrophic and eutrophic water. Error bars denote the standard deviation and *R* ^2^ and *p* values for OLS linear regressions are shown. The dashed lines represent the relative number of OTUs with rrn 1–5 and 9 occurring at each time point. Standard deviation envelopes were calculated from *n* = 3 independent replicates

Our approach allowed us to incorporate growth strategies into the genomic framework that we previously used to characterize the community succession of stream biofilms.^22^ We found that OTUs with two rrn copies were mostly assigned to *Sphingomonas* and *Caulobacter* and dominated throughout biofilm succession independent of trophic state and hydraulics (i.e., laminar vs. turbulent flow) (Fig. 2; Supplementary Figure 3). *Sphingomonas* is known to secrete copious EPS, mostly gellan, to enhance cell adhesion.^28^ *Acidovorax* (Betaproteobacteria; rrn = 3), *Sphingobium* (Alphaproteobacteria; rrn = 3) and members of *Bacteroidetes*, particularly *Flavobacteriales* (rrn = 5), were more abundant during early succession. The gliding motility and elevated growth rates of *Flavobacteriales* likely facilitated the initial colonization of surfaces and subsequent biofilm formation.^29,30^ The relative abundance of these taxa decreased with ongoing succession independent of the trophic state, while *Azospirillum* and *Clostridium* (both rrn = 9) increased in relative abundance during late succession in the oligotrophic system (*R* ^2^ = 0.65 and *R* ^2^ = 0.81; *p* < 0.01, respectively). In the eutrophic system, taxa with 9 rrn copies emerged during early succession but decreased in abundance thereafter to contribute only marginally to mature biofilms (Fig. 2; Supplementary Figure 3).

In the oligotrophic system, taxa with one copy of the rrn emerged after 2 weeks and increased significantly (*R* ^2^ = 0.56; *p* < 0.05) in relative abundance during biofilm maturation. This increase can be attributed to *Phenylobacterium*, the most abundant taxa with one rrn copy in our dataset. *Phenylobacterium* (*Caulobacteraceae)* is a gram-negative, copiotrophic, non-motile, and slow-growing bacterium with a high nutritional specialization.^31,32^ The increasing importance of *Phenylobacterium* during biofilm succession may be attributed to its motility deficiency potentially restricting its ability as an early colonizer of surfaces.

Our experimental results show that bacteria with two copies of the rrn and therefore with a putatively slow-but-efficient metabolic strategy^11^ initiate biofilm formation in both oligotrophic and eutrophic systems. This contrasts ecological theory predicting community succession to be initiated by fast-but-inefficient early colonizers followed by slow-but-efficient secondary colonizers.^33^ The patterns reported here enlarge the repertoire of microbial adaptation strategies reported from soils^15,34^ and sediments^9^ where bacteria with 4 to 9 rrn copies typically initiate community succession. The temporal trajectories of rrn distribution in stream biofilm also differ from bacteria colonizing model marine particles where slow-but-efficient secondary colonizers consume resources produced by fast-but-inefficient early colonizers.^35^ We propose that different ecological strategies and metabolic trade-offs underpin these varying patterns. Initial biofilm formation requires cell adhesion, surface conditioning and EPS production, all of them being energetically expensive processes.^1,2,36^ The observed dynamics in rrn copy numbers under oligotrophic conditions may reflect a metabolic trade-off where a noticeable part of the energy is invested in processes related to biofilm formation rather than in cellular growth. An initially prioritized investment into EPS production is evolutionary justified given the fitness advantages for bacteria to live embedded in the EPS matrix.^2^ The successional patterns observed during biofilm formation under oligotrophic conditions may therefore reflect a colonization-competition trade-off^37^ rather than a rate-yield trade-off.^11^ In streams that are poor in resources, colonizers with low rrn copies (e.g., 2) may efficiently use resources to initiate biofilm formation, while superior competitors with elevated rrn copies (e.g., 9) appear only once relatively stable and productive niches have established within biofilms.^38^ We acknowledge that these findings rest on the abundance of 16S rRNA marker genes and that the actual phenotypes of the bacterial taxa involved in biofilm formation in our experiments are not known.

### Alternative trade-offs in highly fluctuating environments

**Fig. 3:**
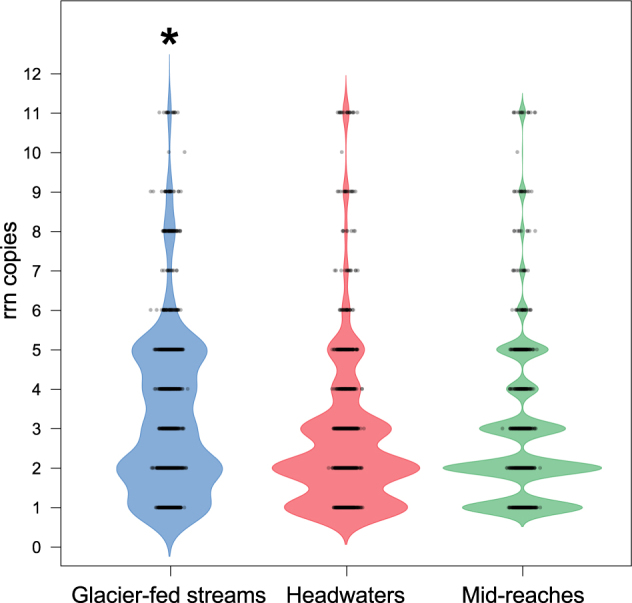
rrn copy number distribution in biofilms from different streams. The rrn probability density distributions (colored areas) of core stream biofilm OTUs (glacier-fed streams = 1825 OTUs; headwaters = 1478 OTUs; mid-reaches = 1240 OTUs) are shown. Underlying box-whisker-plots indicate the median (line), interquartile range (box) and extreme values (whiskers). Asterisks highlight significant differences in median rrn between biofilms from glacier-fed streams and subalpine streams without glacier influence (Mann–Whitney *U*-test, *p* < 0.001)

Next we expanded our experimental findings to benthic biofilms from 140 streams including glacier-fed streams^24^ and subalpine streams with no glacier influence^6^ (See Methods section). We explored whether rrn copy numbers reflect ecological strategies of biofilms to cope with the natural environment in these ecosystems. We compared rrn copy number distribution of OTUs associated with the 182 core biofilm genera among stream types (Fig. 3) (see Methods section). OTUs with two rrn copies dominated in all biofilms but average rrn copy number was significantly higher in biofilms from glacier-fed streams than from subalpine streams (Mann–Whitney *U*-test; *p* < 0.001) (Fig. 3). This difference could be associated with the increasing relative abundance of OTUs affiliated with *Gammaproteobacteria* (rrn 4–12), *Betaproteobacteria* (rrn 4–6), *Firmicutes* (rrn 4–15), and *Flavobacteriales* (rrn 5) in the glacier-fed streams. Although some of these OTUs (e.g., members of the *Firmicutes* and *Gammaproteobacteria*) may be rare, they often comprise a considerable part of the metabolically active fraction of the biofilm community.^25^ Moreover, several of these are spore-forming taxa and therefore capable to resist environmental challenges, such as temperature fluctuations, UV-irradiation and desiccation.^39^ Biofilms from subalpine streams were dominated by *Alphaproteobacteria*, notably *Sphingomonas* (rrn = 2) and *Bacteroidetes*, in particular *Flavobacteriales* (rrn = 5), which were also abundant in the laboratory-grown biofilms described above.

The prevalence of HCN in oligotrophic glacier-fed stream biofilms suggests different trade-offs potentially overruling rate-yield trade-offs in these natural environments. We suggest that short lag phases combined with high growth rates, possibly accompanied with elevated EPS production, are traits beneficial for biofilms to thrive in glacier-fed streams where the environment is highly unsteady during snow- and icemelt.^4,40^ This notion is supported by observations that cell-specific EPS declines with decreasing environmental fluctuations downstream from glaciers.^41^

### rrn copy number distribution is paralleled by functional trait allocation

**Fig. 4:**
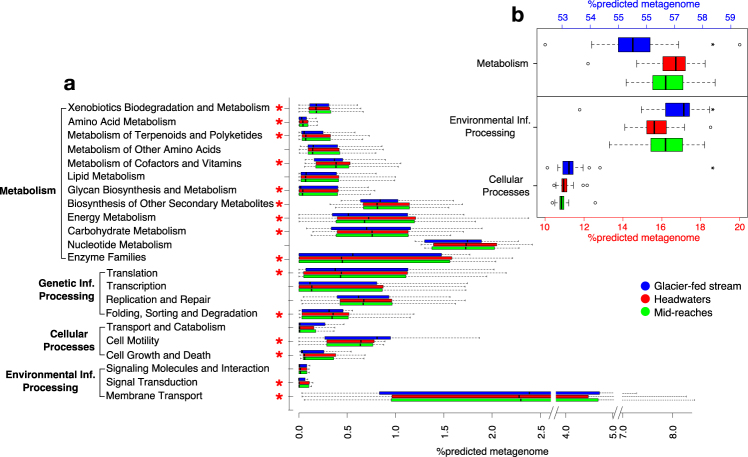
Predicted relative gene family abundances for stream biofilms in different habitats. **a** Boxplots displaying the relative gene counts corresponding to KEGG orthology families from category 2 of biofilms from glacier-fed streams (blue), headwaters (red) and mid-reaches (green). Asterisks (red) indicate gene categories that are significantly different (ANOVA, Tukey HSD, Bonferroni-corrected *p* < 0.05) between biofilms from glacier-fed streams and subalpine streams. Boxes indicate the interquartile ranges, medians are shown as lines within the boxes, whiskers display lower and upper quartiles. **b** Boxplots displaying the relative gene counts corresponding to the KEGG orthology families Metabolism (shown on upper *y*-axis, blue), Environmental Information Processing and Cellular Processes (both shown on lower *y*-axis, red). Boxes indicate the interquartile ranges, medians are shown as lines within the boxes, whiskers display lower and upper quartiles, outliers are shown as circles. Asterisks (black) highlight significant differences in gene counts between biofilms from glacier-fed streams and subalpine streams without glacier influence (ANOVA, Tukey HSD, *p* < 0.01)

To further explore ecological lifestyles related to rrn copy numbers in the biofilms from the glacier-fed and subalpine streams, we inferred putative bacterial metagenomes using PICRUSt^42^ (see Methods section). Predicted genomic features, such as motility, specialized transporters for nutrient acquisition and signal transduction indicate a fast-but-inefficient lifestyle^18^ and prevailed in the biofilms from glacier-fed streams (Fig. 4a). On the other hand, predicted genetic features of biofilms from subalpine streams were characterized by multifunctional transporters for nutrient acquisition, increased production of secondary metabolites, translating into a slow-but-efficient lifestyle.^18^ This impacted on the relative predicted counts from higher ranked gene categories categorized as environmental information processing (ANOVA, Tukey HSD, *p* < 0.001) and cellular processes (ANOVA, Tukey HSD, *p* < 0.001), which were more abundant in biofilms from glacier-fed than from subalpine streams (Fig. 4b). Relative predicted gene counts categorized as metabolism, in contrast, were significantly less abundant in biofilms from glacier-fed than from subalpine streams (ANOVA, Tukey HSD, *p* < 0.001) (Fig. 4b). Despite the uncertainty of metagenome predictions from 16S rRNA genes as inherent to PICRUSt,^42^ these patterns support our observations on growth-related strategies based on experiments and rrn copy numbers.

In summary, our findings unravel ecological strategies underlying the success of biofilms in stream ecosystems. Such strategies are in line with streamlining theory^20^ and variation between alternative trade-offs may explain community succession trade-offs in complex biofilm communities. Our findings provide genomic underpinnings of biofilm ecology and expand current knowledge on biofilm formation hitherto largely derived from mono-species systems grown on the laboratory bench.

## Methods

### Material

We used three previously published datasets of 16S rRNA gene amplicon sequences from stream biofilms. Because these datasets were produced by the same research group, molecular techniques and bioinformatics pipelines were comparable. We used data from an experiment using benchtop flumes to study biofilm formation and community succession.^22^ Biofilms were grown (45 days) from oligotrophic streamwater (concentration of dissolved organic carbon, DOC: 1.7 ± 0.2 C mg L^−1^; concentration of nitrate: 0.52 ± 0.01 NO_3_–N mg L^−1^) and eutrophic river water (DOC concentration: 2.6 ± 0.9 mg L^−1^, concentration of nitrate: 1.1 ± 0.1 NO_3_–N mg L^−1^), respectively. Next, we re-analyzed sequences from benthic biofilms from 114 subalpine streams.^6^ These samples were split into headwaters (1st to 3rd stream order; catchment area <5 km^2^; *n* = 50) and mid-reaches (2nd to 5th stream order; catchment area >5 km^2^; *n* = 64). These subalpine streams had no glacier influence. Furthermore, we re-analyzed sequences from biofilms sampled from 26 glacier-fed streams.^24^ All OTU abundance tables were rarified prior to analysis. We selected those genera (*n* = 182) that were present in all biofilm samples (experiments, subalpine, and glacier-fed streams), thus reflecting a core stream biofilm community. In total, 1478 OTUs for headwaters, 1240 OTUs for mid-reaches, 1825 OTUs for glacier-fed stream biofilms and 5130 OTUs for laboratory derived biofilms corresponding to these 182 core genera were included (Supplementary Figure 1). OTUs corresponding to these core genera comprised 95% of all OTUs found in glacier-fed stream biofilms, 30% from headwater streams, 31% from mid-reaches, and 77% from biofilms derived from the experiments.

### Estimation of the ribosomal RNA operon (rrn) copy number

We estimated rrn copy numbers for OTUs in our datasets by querying the rrnDB^26^ (version 4.43) on genus level. The rrnDB database includes 2865 bacterial records derived from annotations of published genome sequences. Estimated rrn copy numbers were rounded to the next integer as rrnDB provides averages.

### Data analyzes and statistics

We summed up the relative abundances of OTUs with low rrn copy numbers (1–3, LCN) and of OTUs with high rrn copy numbers (4–15, HCN) and calculated the community-aggregated ratio of HCN to LCN (HCN:LCN) for each time point during biofilm succession. We then computed the average ratios over all time points and related them to the corresponding maximum growth rates (*µ*_max_) and lag phases (*L*), respectively. *µ*_max_ and *L* were obtained from fitting a logistic growth model to microscopic counts.^22^

We calculated the average rrn copy number for the shared genera in all our samples as the pool of potential migrants and randomly sampled 150 OTUs from this community 1000 times. The average rrn copy number from these random samples was compared with the average rrn copy number during biofilm formation to exclude a statistical bias through the rrn distribution of the pool of potential migrants (Supplementary Discussion). Furthermore, the relative number of genera contributing to HCN and LCN were compared between biofilms from oligotrophic and eutrophic biofilms.

All analyzes were conducted using R^43^ and the packages grofit^44^ and ggplot2.^45^ We used PICRUSt (Phylogenetic Investigation of Communities by Reconstruction of Unobserved States)^42^ to analyze putative metagenomic differences of the biofilm core communities grown in different environments. We followed the suggested methods for OTU picking with Greengenes 13.5 using Galaxy (http://huttenhower.sph.harvard.edu/galaxy/). PICRUSt then transformed counts of reference-based OTUs into counts of functional genes specified by KEGG (Kyoto Encyclopedia of Genes and Genomes) orthology groups. The relative contribution of identified microbial gene families (i.e., Metabolism, Environmental Information Processing and Cellular Processes) was compared using STAMP.^46^

### Data availability

The raw 16S rRNA gene sequences analyzed in this study are available at the public NCBI Sequence Read Archive (SRA) under the accession numbers SRX196420,^24^ SRP076464,^6^ and SRP076464.^22^ Auxiliary data analyzed during the current study are available from the corresponding author on request.^43,45,46^

## Electronic supplementary material

Supplementary Material
