# Supplementary material for: Ecological strategies and metabolic trade-offs of complex environmental biofilms
Source: NPJ Biofilms Microbiomes. 2017 Sep 25;3:21. doi: 10.1038/s41522-017-0029-y (PMC5612939; doi:10.1038/s41522-017-0029-y)
Supplement: Supplementary file 1 — Supplementary Material [file 41522_2017_29_MOESM1_ESM.pdf]

Ecological strategies and metabolic trade-offs of complex environmental biofilms

Robert Niederdorfer[a,b], Katharina Besemer[c], Tom J. Battin[a], Hannes Peter[a*]

[a] Stream Biofilm and Ecosystem Research Laboratory, School of Architecture, Civil and Environmental Engineering, École Polytechnique Fédérale de Lausanne, Lausanne, Switzerland

[b] Department of Limnology and Oceanography, University of Vienna, Vienna, Austria

[c] Wasser Cluster Lunz, Lunz am See, Austria

* corresponding author: hannes.peter@epfl.ch

This file includes:

Supplementary Figures 1-3
Supplementary Discussion

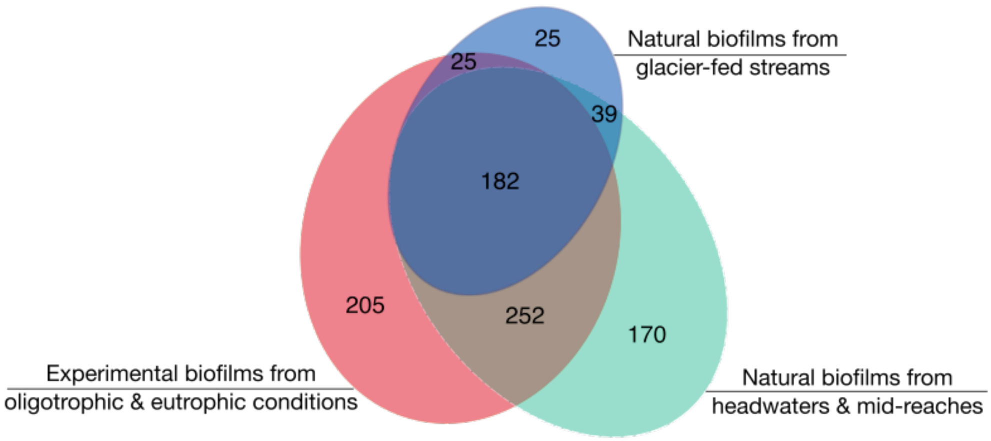

Supplementary Figure 1 Venn diagram highlighting the number of bacterial genera from biofilms overlapping between the different datasets. We consider these 182 genera as typical biofilm formers in streams.

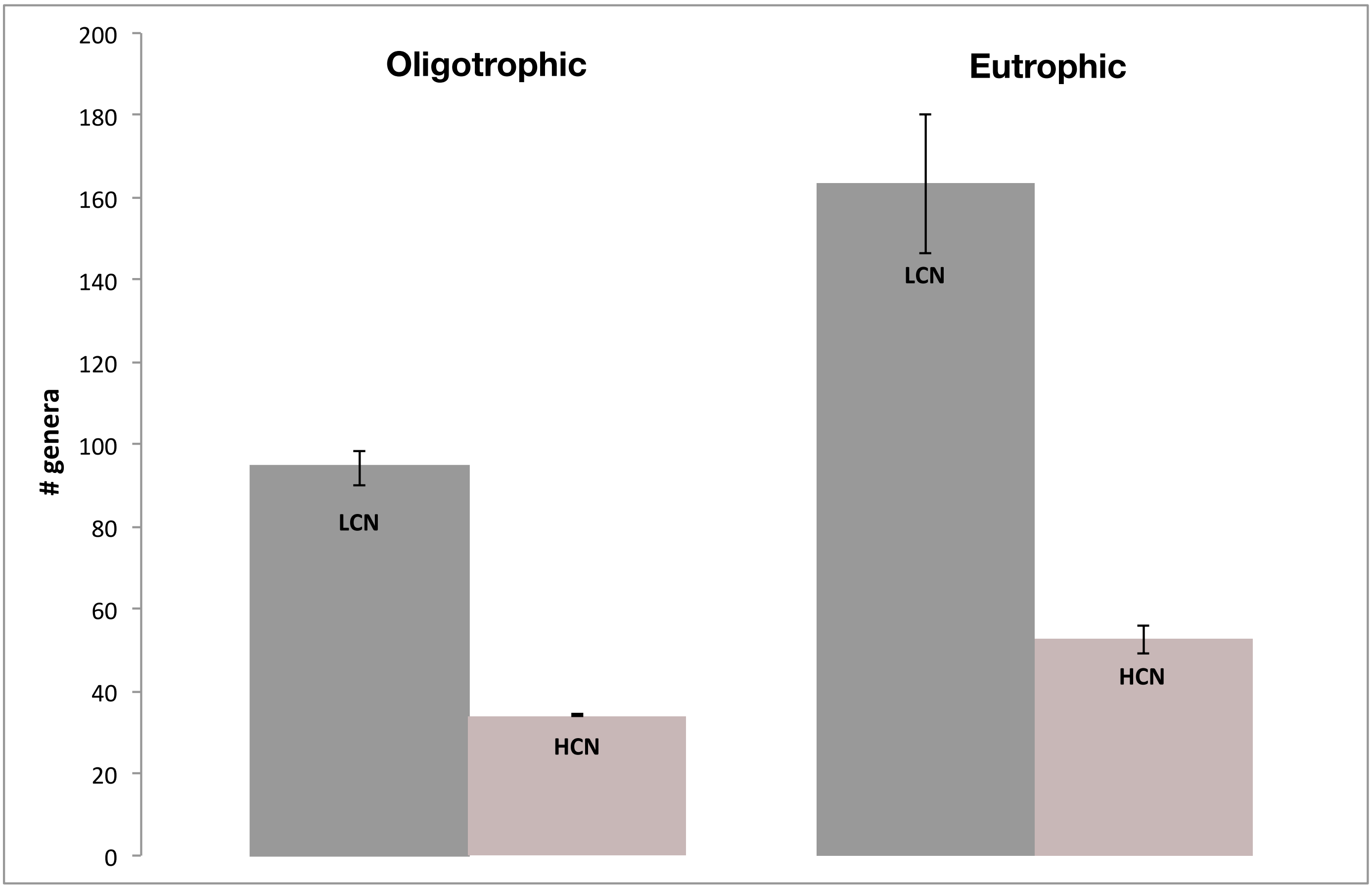

Supplementary Figure 2 Average number of genera within rrn categories (LCN, HCN). Error bars denote the standard deviation (n=9).

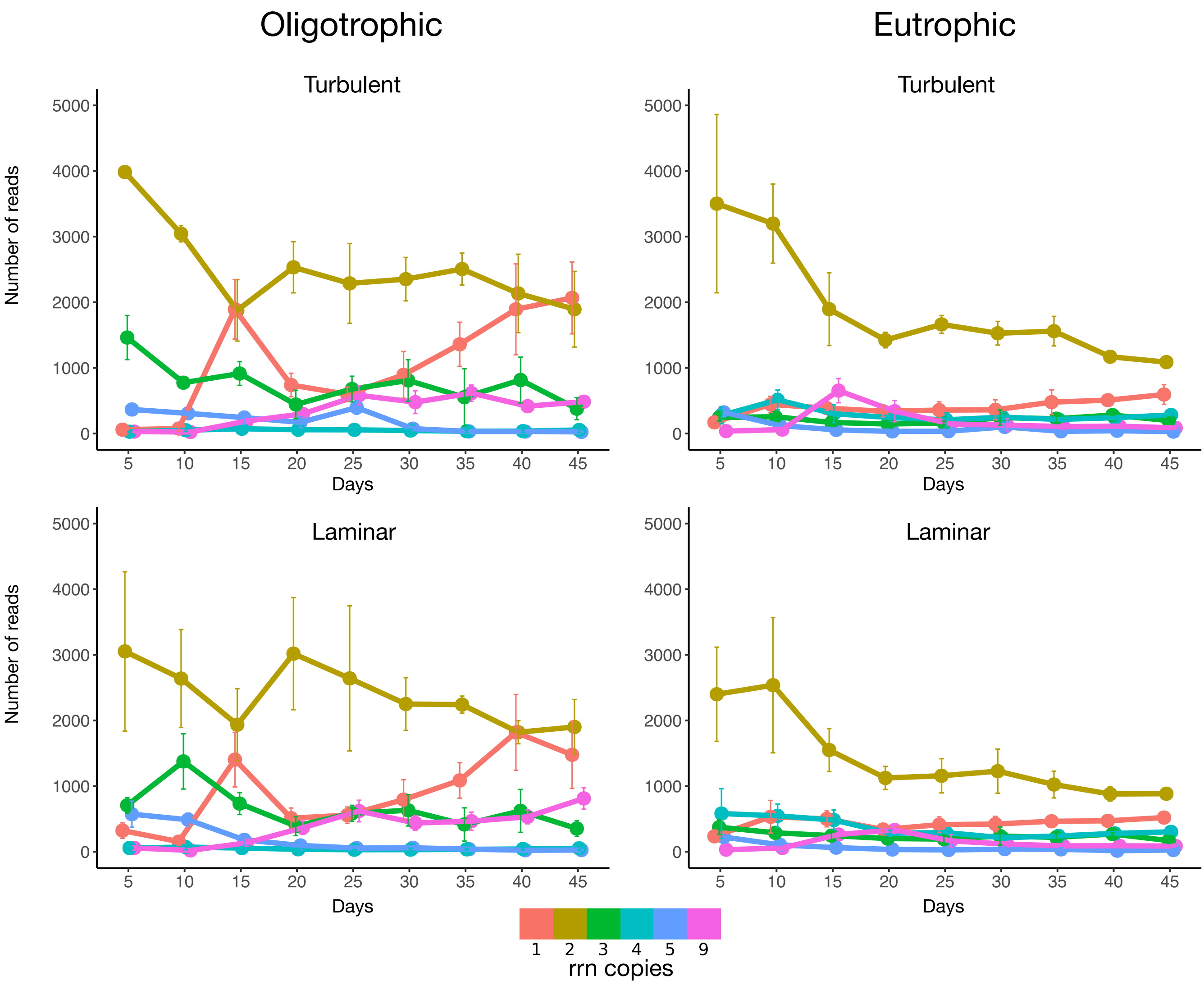

Supplementary Figure 3 Temporal dynamics of OTUs categorized according to rrn copy number. Shown are the temporal dynamics of absolute read counts in order to exclude the possibility that observed temporal dynamics of rrn groups during biofilm formation were merely caused by changes in relative abundance of one community member affecting the relative abundance of other members (Figure 2 in the main text). Lines denote the average absolute read counts of OTUs with rrn 1-5 and 9. OTUs with rrn 6-8 and >9 were rare and did not contribute to stream biofilm communities originating from oligotrophic and eutrophic water. Error bars denote the standard deviation of n=3 replicates.

Supplementary Discussion:

In general, there are more bacteria with few rrn copies than there are with many rrn copies in their genomes. Assuming that this distribution affects the likelihood to be samples from the potential pool of migrants into the biofilms, this could result in a statistical bias of under-sampling taxa with high rrn copy numbers. To exclude the possibility that the temporal patterns in rrn copy numbers that we observed during biofilm succession are due to such a systematic under-sampling, we contrasted our results random samples from the possible pool of migrants. We considered the 5130 OTUs in 182 genera common to all samples as the potential pool of migrants in stream biofilms and estimate an average rrn copy number of 3.4. We then randomly sampled 150 OTUs from this pool of migrants, since on average 126 OTUs were present in oligotrophic samples and 198 OTUs in eutrophic samples. We repeated this random sampling 1000 times. Average rrn copy numbers ranged between 2.5 and 3.5 with an average rrn copy number of 3.02 ± 0.18 for a random sample from the potential pool of migrants of a similar size as the communities observed in our experiments. Average rrn copy number was 3.16 ± 0.15 for biofilms grown under oligotrophic conditions and 3.05 ± 0.11 for biofilms grown under eutrophic conditions and well within the range of potential migrants. This shows that the rrn copy number distribution of the potential pool of migrants does not explain the temporal patterns in rrn copy numbers that we observed in our experiments.
